# Reduced Predicted Glycaemic Response and Inhibitory Effects on Lipid-Digesting Enzymes of Pasta Enriched With Flour of *Malus domestica* “*Annurca* cv.” Pulp or Peel

**DOI:** 10.1155/ijfo/8361330

**Published:** 2025-04-24

**Authors:** Maria Neve Ombra, Filomena Nazzaro, Florinda Fratianni

**Affiliations:** National Research Council, Institute of Food Science, Avellino, Italy

**Keywords:** enriched pasta, lipid-digesting enzymes, low predicted glycaemic index (pGI)

## Abstract

Pasta is widely consumed, and incorporating certain components can transform it into a functional food with health advantages. These components include antioxidants, dietary fiber, and enzyme inhibitors associated with a decreased risk of various health issues. The *Malus domestica* variety *Annurca* is abundant in bioactive compounds, making it a suitable candidate for producing functional foods. We developed durum wheat tagliatelle enriched with dried and powdered *Annurca* pulp or peels at two distinct supplementation levels (3% and 6%) on a laboratory scale. The in vitro ability to inhibit digestive enzymes and the predicted glycaemic value of the enriched pasta were evaluated. Every formulation demonstrated inhibition of *α*-amylase, *α*-glucosidase, cholesterol esterase, and lipase in vitro. All the samples exhibited a low predicted glycaemic index (pGI), ranging from 41.25 to 45.76. These low pGI values indicate that fortified pasta has a lower impact on blood sugar levels and a slower digestion rate. The predicted glycaemic indices for pasta enriched with 3% or 6% pulp flour were 18.7% and 26.7% lower, respectively, than those for control pasta with durum wheat semolina alone. The reductions were similar for peel-enriched pasta at 3% and 6% (24.4% and 24.7%, respectively). Our in vitro results indicate that *Annurca* apple-fortified pasta has lipid- and glucose-lowering effects. Adding *Annurca* fruit flour with valuable and functional compounds could be relevant for obtaining pasta with potential health benefits.

## 1. Introduction

Shifts in global food systems, the increasing consumption of high-calorie diets, and sedentary lifestyles have contributed to the rise of obesity and Type 2 diabetes mellitus. Obesity and hyperlipidaemia are key risk factors for diabetes mellitus, making weight management crucial for the effective treatment of diabetes and its related complications. Proper nutrition and wise food choices are vital for maintaining good health [1]. Research increasingly underscores the importance of suitable dietary regimens in preventing and delaying the onset of major aging-related diseases. Among these, the Mediterranean diet, rich in plant-based micro- and macronutrients, has demonstrated significant health benefits, including lipid-lowering and blood sugar–regulating effects attributed to its polyphenolic compounds [2]. These bioactive molecules, abundant in fruits and vegetables, modulate digestive enzyme activity, potentially influencing calorie assimilation and offering a promising approach for obesity-related disease management [3–6]. The inhibition of key enzymes involved in lipid and carbohydrate digestion, such as lipase, cholesterol esterase (CEase), *α*-amylase, and *α*-glucosidase, has emerged as an effective strategy for managing obesity and hyperglycaemia [7, 8].

Lipase and CEase hydrolyse lipid substrates like triglycerides, phospholipids, cholesterol esters, and fat-soluble vitamins [9]. Lipase is particularly crucial for the digestion of dietary fat triacylglycerols into simpler compounds like monoacylglycerols and fatty acids, which are then absorbed in the intestine. CEase is essential for hydrolysing dietary cholesterol esters, facilitating the transfer of free cholesterol from micelles to enterocytes [10], and facilitating its absorption. By inhibiting these enzymes, natural compounds act to slow the rate at which lipids are digested. This moderated digestion results in a more gradual release and absorption of fatty acids, which can help manage overall caloric intake from fats. Although obesity is a multifactorial condition influenced by genetic, environmental, and behavioral factors, this slower digestion of dietary fat can be a complementary strategy to help maintain energy balance and manage weight gain over time. Several natural compounds, including polyphenols, flavonoids, alkaloids, and saponins, have been identified as effective inhibitors of these enzymes, supporting their potential in obesity control [5]. Similarly, pancreatic *α*-amylase and intestinal *α*-glucosidase play key roles in starch digestion and blood glucose regulation. Inhibiting their function can substantially slow the conversion of starch or disaccharides into monosaccharides, significantly reducing postprandial blood sugar levels.

Currently, popular foods are being transformed into carriers of functional ingredients that offer nutritional and health benefits [11, 12]. Among popular foods, pasta satisfies nutritional needs and offers various enrichment options for preventing or alleviating diet-related disorders [13]. Pasta is a widely consumed staple food across the globe, making it an ideal vehicle for incorporating bioactive compounds. Enriching pasta with vegetables results in a high-quality product from a nutritional standpoint, offering numerous health benefits. It can be readily enriched with plant-based materials containing bioactive compounds that possess antioxidant, hypoglycaemic, or cholesterol-lowering properties [14]. Fortified pasta with a low glycaemic response can be inserted into diets aimed at glucose control. Studies have indicated that traditional pasta made from durum semolina has a glycaemic index (GI) spanning from 43 to 60 [15, 16]. Moreover, low GI foods are indicated for diabetic patients as they reduce postprandial hyperglycaemia [17, 18]. The inclusion of active molecules and fibers that interact with starch affects carbohydrate digestion and absorption, which is particularly significant for diabetes management. Various studies have shown that compounds such as flavonoids, anthocyanins, phenolic acids, saponins, carotenoids, terpenes, and plant proteins exhibit *α*-amylase and *α*-glucosidase inhibitory capacities [19].

*Malus pumila Miller* cv. *Annurca* is a cultivar autochthon of Southern Italy, registered on the list of Protected Geographical Indication (PGI) products of the European Council (C. R. 0[EC]No.417/2006) [20]. *Annurca* cv. is white and crisp, has decidedly firm flesh, is exquisitely aromatic, and has a pleasantly acidulous and fragrant flavour, unlike other cultivars. It grows under special climatic conditions, undergoing a particular reddening treatment. First, the apples do not ripen on the tree but are harvested still unripe, in autumn. Then, they are placed on typical straw beds for approximately 1 month. In this way, they can ripen slowly in the sun, and the straw serves as insulation to absorb any moisture. The fruits are continuously turned over so that the greenest parts are exposed to the sun until the whole fruit acquires its typical bright red colour. Due to this process, the *Annurca* apple acquires the characteristic aroma and flavour [21].

Polyphenols and triterpenes are secondary metabolites frequently present in apple varieties. Identified proanthocyanidins included catechin, epicatechin, and procyanidin, while the recognized flavonols encompassed kaempferol, quercetin, rutin, phloretin, and phloridzin. In addition, chlorogenic, ferulic, *p*-coumaric, caffeic, and gallic acids were found [21–23]. Triterpenes in the peel mainly included oleanolic, betulinic, ursolic, and annurcoic acids; the latter was so named because it was first highlighted in the *Annurca* cultivar [24]. Our previous study revealed that epicatechin, rutin, caffeoylquinic acid, and chlorogenic acid were the predominant phenolic compounds in the peel. Overall, the *Annurca* apple variety is richer in catechin, epicatechin, chlorogenic acid, and procyanidin B2 than other varieties [20, 21]. Polyphenols from the *Annurca* cultivar have been extensively studied for their ability to regulate cholesterol levels and mitigate metabolic syndrome. Randomized clinical trials have confirmed the efficacy of both the whole fruit and its aqueous extracts in improving metabolic health [25, 26].

Aligned with the principles of a circular economy, there is an increasing interest in using food industry by-products as sources of beneficial compounds such as antioxidants and dietary fibers [27]. One promising by-product in this regard is the apple peel. The peel of the *Annurca* apple, which constitutes about 20% of the fruit's total mass, with an estimated annual production of over 60,000 tons of *Annurca* apple is a cost-effective source of phenolic compounds and dietary fiber that promote satiety, intestinal health, and cholesterol reduction. Despite the recognized health benefits of dietary fiber [28], many individuals fail to meet the recommended daily intake of 25–30 g, highlighting the need for fiber-enriched foods. To our knowledge, no investigations have examined the effects of *Annurca* flour in pasta on enzyme inhibition, particularly *α*-amylase, *α*-glucosidase, lipase, and CEase. Our research aimed to evaluate the impact of adding 3% and 6% *Annurca* pulp or peel powder on the functional properties of pasta, exploring its potential as a functional food with hypoglycaemic and lipid-lowering properties.

## 2. Methods

### 2.1. Materials

Pancreatic *α*-amylase (A 6255), yeast *α*-glucosidase (G5003), porcine pancreatic pepsin (P7545), pancreatic lipase (L3126), pancreatic CEase (C23766), acarbose (A8980), orlistat (O4139), dinitrosalicylic acid (DNSA) colour reagent (D0550), p-NPB (*p*-nitrophenyl butyrate), taurocholate salt, *p*-nitrophenyl-*α*-d-glucopyranoside (N1377), and gallic acid (8426490025) were procured from Merck KGaA, Darmstadt, Germany. Acetone, hydrochloric acid, sodium hydroxide, ethanol, and sodium carbonate were bought from CARLO ERBA Reagents, Italy. Durum wheat semolina (Cappelli), Mela *Annurca* PGI, and bread wheat flour (Selex, Trezzano sul Naviglio, Milan, Italy) were obtained from a local store in Avellino (Italy).

### 2.2. Preparation of Apple Powders and Extracts

The apples were washed with distilled water. The pulp and peel were separated, sliced, and dried at 50 ± 2°C using a tray dryer (Melchioni Babele 250 W, five shelves) for 10 h. After drying, the materials were milled (Kenwood CH580) and sifted through a 500-*μ*m sieve to obtain apple powders. These powders were either stored in sealed dark bags for further analysis or added to wheat flour at two concentrations (3% or 6%), for the dough preparations.

The powders were extracted with 50% aqueous ethanol (1.0 g/10 mL; w/v). The samples were shaken 24 h at 20°C and then centrifuged at 13,000 g for 5 min [29]. The resulting supernatants were used for in vitro assays.

### 2.3. Pasta Preparation

Four types of tagliatelle enriched with apple pulp or peel powders and one control sample (Ctrl) were prepared. In brief, apple powder (7.5 g or 15 g) and durum wheat semolina (242.5 g or 235 g), for 3% and 6% formulation, respectively, were combined with 100 mL of tap water to form the dough. The moisture content of the resulting dough was about 32%, and it was extruded into tagliatella shape, 2.0 mm thick and 20 cm long, with pasta maker (Ariete Pastamatic 1593) and labelled as AP3, AP6 (*Annurca* peel powder), and AF3, AF6 (*Annurca* flesh powder). After cooking, the fortified tagliatelle were named AP3c, AP6c, and AF3c, AF6c. The control pasta (Ctrl) was made using 100% durum wheat semolina. After 48 h at 25°C, the samples were conserved in sealed bags, at room temperature, until used.

### 2.4. Cooking Time Determination and Cooking Performance

Optimal cooking time (OCT) was calculated using the AACC-approved method 66-50, as described by Samaan et al. [30], and resulted to be 10 min. Ten grams of tagliatelle was boiled in 100 mL of water. Every 60 s, a small portion was divided in half widthwise and pressed between two glass slides. The cooking time was recorded when the opaque, uncooked center of the pasta, known as the white starchy core, had completely vanished, signifying that the starch was fully cooked and evenly absorbed water. Samples of 10 g of pasta were boiled in 500 mL of water for 10 min, washed with 100 mL of cold water, and strained for 30 s to evaluate cooking loss (CL), swelling index (SI), and water absorption index (WAI).

CL was measured following AACC guidelines, where the cooking water was collected in an aluminum container and dried in an air oven at 105°C until a constant weight was achieved. The remaining residue was weighed and expressed as grams per 100 g of uncooked pasta [31].

The SI and WAI were determined based on a modified version of the method by Desai et al. [32]. After cooking, 10 g pasta samples were weighed after washing and straining (Pc), then dried at 105°C until a constant weight was reached (Pd). The SI was calculated using the following formula:

 $$\begin{array}{c} \text{SI}=\left(\text{Pc}-\text{Pd}\right)/\text{Pd} \end{array}$$

The WAI was calculated using the following equation:

 $$\begin{array}{c} \text{WAI}=\left(\text{Pc}-\text{Pu}\right)/\text{Pu}\times 100 \end{array}$$

where Pu is the weight of uncooked pasta, Pc is the weight of cooked pasta, and Pd is the weight of dried cooked pasta. All tests were conducted in triplicate.

### 2.5. Total Phenol Content

The total polyphenol content (TPC) was measured following the method described by Ombra et al. [33] and reported as gallic acid equivalent (GAE) in micrograms per gram of sample.

### 2.6. Polyphenols Extraction From Pasta Samples

Polyphenols were extracted from pasta samples according to the protocol outlined by Bustos et al. [34] with modifications. Initially, 5 mL of 70% acetone (in H_2_O) was added to each gram of pasta and stirred for 2 h at 26°C. The mixture was then centrifuged at 10,000 g for 12 min, and the supernatants were collected. This extraction process was repeated, and the supernatants were united. The solvent was removed using a rotary evaporator, and the dried residue (30 mg) was dissolved in water for successive assays.

### 2.7. Alpha-Amylase Inhibition Assay

The *α*-amylase assay, adapted from the Sigma-Aldrich protocol with modifications [33], was conducted with the appropriate negative controls to ensure accuracy. A mixture of 20 mL of 96 mM DSN solution (3,5-DNSA), 8 mL of sodium potassium tartrate (5.31 M) in 2 M NaOH, and 12 mL of deionized water was prepared as the reaction mixture. Porcine pancreatic *α*-amylase was dissolved in 20 mM phosphate buffer (pH 6.9) containing 6.7 mM NaCl. Ninety microliters of the sample was added to 10 *μ*L of amylase solution (575 U/mL) and incubated at 25°C for 20 min. A 1.0% starch solution (100 *μ*L) was then added and incubated at 25°C for 3 min. Subsequently, 100 *μ*L of DNS reagent was added; the mixture was incubated at 85°C for 10 min and later diluted with 1.0 mL of deionized water. Negative controls were performed by substituting the extracts with 90 *μ*L of distilled water, to verify the specificity of the reaction. Blank samples were prepared by including the extract in the reaction mixture without *α*-amylase enzyme. The absorbance at 540 nm was measured, and the blank value was subtracted from the sample absorbance. Acarbose (1.0 mM) served as the positive control. The IC50 value, that is, the concentration of the extract that causes 50% inhibition, was determined based on three separate experiments. For pasta extracts, % inhibition was calculated using the following formula:

 $$\begin{array}{c} \%\text{enzyme inhibition}=1-\text{Asample}/\text{Acontrol}\times 100\% \end{array}$$

where Asample is the absorbance of the extract and Acontrol is the absorbance of the control (without inhibitors).

### 2.8. Alpha-Glucosidase Inhibition Assay

Following the method by Sharp et al. [35], 10 *μ*L of extract and a 5 mg/mL solution of *α*-glucosidase from *Saccharomyces cerevisiae* (10 *μ*L), 1 mM solution of *p*-nitrophenyl-*α*-d-glucopyranoside (25 *μ*L) in 20 mM phosphate buffer (pH 6.0), were incubated at 37°C for 10 min. After incubation, 80 *μ*L of 0.1 M Na_2_CO_3_ solution was added, and the absorption was measured at 400 nm. Acarbose (1.0 mM) served as the positive control. The IC50 value was determined through three repeated experiments. For pasta extracts, *α*-glucosidase inhibition activity was indicated as percent inhibition, calculated using the following formula:

 $$\begin{array}{c} \%\text{inhibition}=1-\text{Asample}/\text{Acontrol}\times 100\% \end{array}$$

where Asample is the absorbance of extract and Acontrol is the absorbance of the control (without inhibitors).

### 2.9. Pancreatic Lipase Inhibition Assay

The inhibition capacity of extracts against pancreatic lipase was assessed in 96-well plates, as described by Ombra et al. [33]. Porcine pancreas Type 2 lipase was dissolved in ultrapure water at 2.5 mg/mL, centrifuged at 2000 g for 6 min, and the supernatant was used immediately. A mixture of 40 *μ*L enzyme solution, 40 *μ*L extract solution, and 20 *μ*L of 10 mM p-NPB substrate solution was incubated for 10 min at 37°C. The absorbance was read at 405 nm. A solution of 1 mg/mL of orlistat was dissolved in DMSO, then diluted with phosphate assay buffer to minimize the final concentration of organic solvent, stirred for 5–10 min, and used as the positive control.

### 2.10. CEase Inhibition Assay

CEase inhibitory activity of extracts was measured following Ngamukote et al. [36] with some changes. Various concentrations of sample extracts (50 *μ*L) were mixed with 50 *μ*L of p-NPB solution (1 mg/mL in 100 mM NaCl, 5 mM sodium taurocholate, 100 mM sodium phosphate buffer, and pH 7.0) and incubated at 37°C for 10 min. CEase solution (50 *μ*L, 0.16 U/mL in 0.1 M sodium phosphate, and pH 7.0) was then added, followed by incubation at 37°C for 20 min. The absorbance was read at 405 nm. The inhibition rate was measured using the following formula:

 $$\begin{array}{c} 1-\text{Asample}–\text{Ablank}/\text{Atest}–\text{Acontrol}\times 100\% \end{array}$$

where Asample is the absorbance of the extract, CEase, and p-NPB; Ablank is the absorbance of extract, p-NPB, and buffer (50 *μ*L); Atest is the absorbance of CEase, p-NPB, and distilled water (50 *μ*L); and Acontrol is the absorbance of p-NPB, distilled water (50 *μ*L), and buffer (50 *μ*L).

### 2.11. In Vitro Starch Digestibility and Predicted GI (pGI) Calculation

In vitro simulated starch digestion was conducted using the method of Brennan and Tudorica [37], with changes as reported in Ombra et al. [38]. Cooked tagliatelle (4 g) was mixed with 20 mL of sodium phosphate buffer (pH = 6.9), adjusted to pH 1.5 with hydrochloric acid for proper porcine pancreatic pepsin activity. Four milliliters of porcine pancreatic pepsin (115 U/mL) was added, and the reaction mix was incubated at temperature of 37°C for 30 min. The pH was then adjusted to 6.9 with sodium hydroxide (2 M), and 1 mL of porcine pancreatic *α*-amylase (110 U/mL) was added to the final 50 mL reaction mixture. The mixture was incubated in the stirred water bath at the same temperature of 37°C. At each time interval (5, 10, 15, 20, 30, 60, 90, 120, 180, and 240), 400 *μ*L of stop solution (0.3 M Na_2_CO_3_) was added to 100 *μ*L aliquot to halt enzyme activity. Aliquots were centrifuged at 2000 g for 5 min, and the 3,5-DNSA method was used to determine reducing sugar quantity at 546 nm [39]. White wheat bread, without inhibitors, served as the reference for the starch digestion rate. The method described by Brennan and Tudorica [40] was adopted to measure the amount of reducing sugar (maltose) released (%RSR) (Equation (1)), hydrolysis index (HI) (Equation (2)), and pGI (Equation (3)).

(1) $$\begin{array}{c} \%\text{RSR}=\left(\text{Asample}\times 500\times 0.95\right)/\left(\text{Amaltose}\times \text{carbohydrate}\right)\times 100 \end{array}$$

Asample is the absorbance at 546 nm; Amaltose is the absorbance of maltose solution (1 mg/mL) by enzyme starch hydrolysis; carbohydrate is the starch and sugars in a 4 g sample.

The HI was calculated as follows:

(2) $$\begin{array}{c} \text{HI}=\text{AUC}\left(0-240\min\right)\text{sample}/\text{AUC}\left(0-240\min\right)\text{bread}\times 100 \end{array}$$

AUC represented the area under the sample curve from 0 to 240 min, normalized to wheat bread.

The pGI was measured using the following equation:

(3) $$\begin{array}{c} \text{Predicted GI}=0.862\text{HI}+8.189 \end{array}$$

### 2.12. Statistical Analysis

Results presented as mean ± standard deviation of three tests were subjected to a statistical analysis. One-way ANOVA (analysis of variance) was applied with a high confidence level (95%, *p* < 0.05) to assess differences between samples. Means were further analysed using student's *t*-test, with significant differences accepted at *p* < 0.05. The dependable tool “Statistics-Excel” was used for these calculations, while IC50 calculations were determined using the trusted online software “ED50plus V1.0” [41].

## 3. Results and Discussion

**Figure 1:**
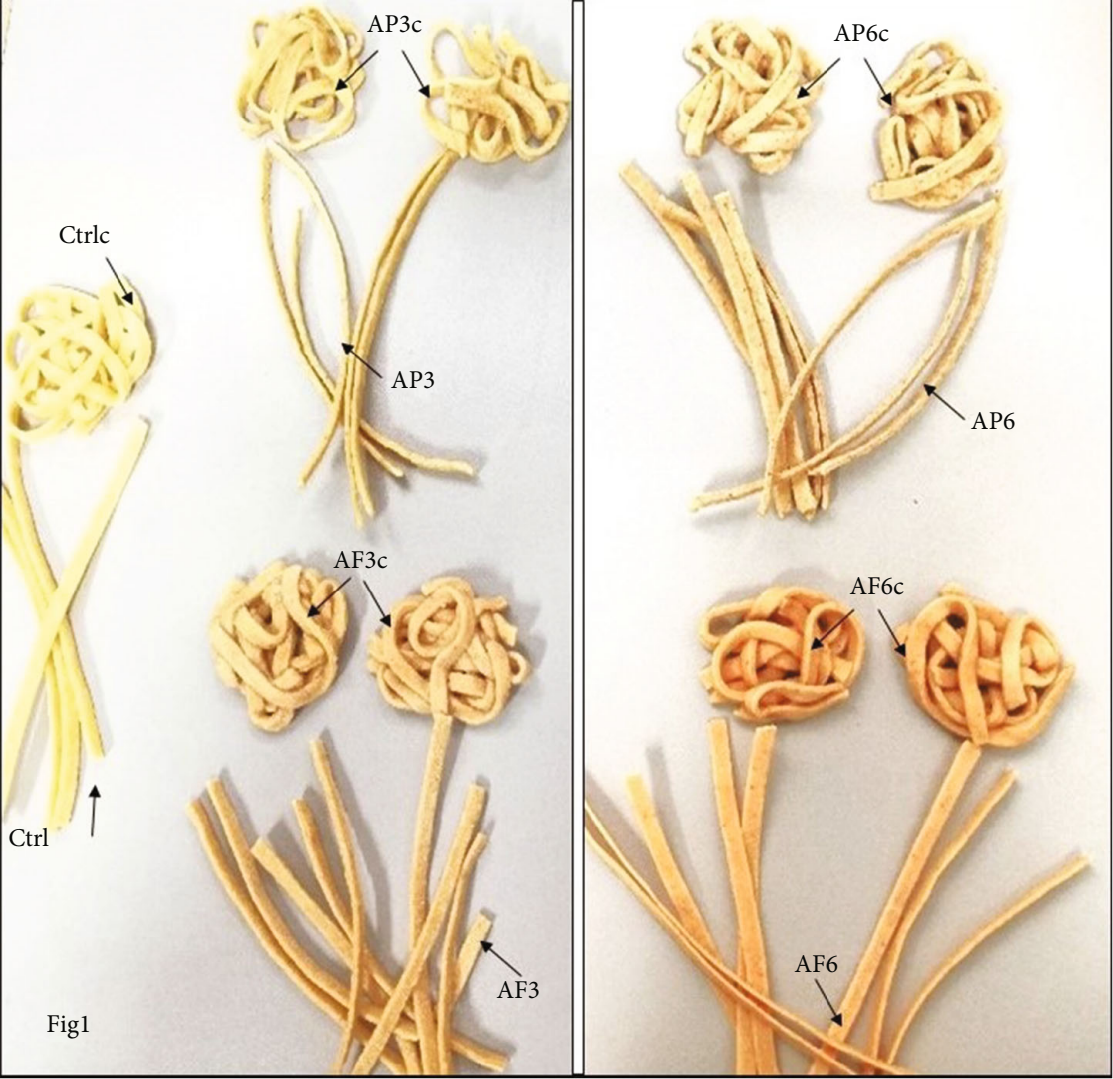
Images of pasta samples. Image of 3% and 6% *Annurca* flesh-fortified tagliatelle, before cooking (AF3–F6) and after cooking (AF3c–F6c), and of 3% and 6% *Annurca* peel-fortified raw samples (AP3–AP6) and cooked samples (AP3c–AP6c). Ctrl = pasta with durum wheat semolina; Ctrlc = cooked pasta with durum wheat semolina.

Apple variety *Annurca*, which has a long history of cultivation in the Campania region of Southern Italy, formed the basis of our investigation. As described in the Methods section, *Annurca* apple pulp and peel powders were incorporated into durum wheat flour (3% or 6%) to produce enriched tagliatelle (AP3, AP6, AF3, and AF6). After cooking, samples were labelled as AP3c, AP6c, AF3c, and AF6c (Figure 1). Initial taste tests indicated satisfactory acceptance overall when up to 6% of the pasta was enriched with apple pulp or peels (data not shown).

### 3.1. Cooking Performance

OCT, CL, SI, and WAI are key indicators of pasta quality during cooking. These factors are largely influenced by the protein–starch network formed during the cold extrusion process. High-quality pasta features a well-structured protein–starch matrix, which slows water penetration into the starch and limits the release of amylose into the cooking water, leading to a longer OCT and reduced CL [31]. The OCT of fortified pasta remained the same as that of the control pasta, with all samples showing a cooking time of 10 min. This could be because at low substitution levels, the gluten network is not sufficiently altered to produce a noticeable effect.  Table 1 reveals that the addition of *Annurca* flour did not significantly reduce CL. Moreover, AP3c and AP6c samples exhibited similar SI and WAI values compared to the control, while the SI and WAI of AF3c and AF6c samples were lower, but not significantly (*p* > 0.05). Previous research has shown that the SI and WAI of vegetable-enriched pasta are influenced by both the integrity and the strength of the gluten network and the water-binding capacity of the vegetable components [42]. The slight differences observed in the values of the samples fortified with *Annurca* peel or pulp in Table 1 may be explained by minimal variations in the water affinity of their components and the strength of the gluten network.

### 3.2. Determination of TPC

To replace durum wheat flour with apple pulp or peel flour, we first measured the TPC of the dried pulp or peel extracts. As indicated in Table 2, the TPCs measured for the peel and pulp extracts were 1.84 mg (GAE)/g DW and 1.23 mg (GAE)/g DW, respectively. The result demonstrated a notable difference between the two concentrations, with the peel extract containing a higher amount of total polyphenols compared to the pulp extract. This finding corroborated our previous studies [20, 21] comparing pulp and peel extracts. Upon replacing semolina with the two flours, a remarkable increase in the polyphenol content of the raw and cooked pasta extracts was observed (*p* < 0.05), surpassing that of the control (Ctrl) pasta. The amount of raw AP6 pasta, for instance, reached a peak of 200.6 ± 6.9 *μ*g GAE/g (Table 2). This enrichment was 5.9 times greater than that of pasta without any addition, indicating a potential boost in health benefits.

Cooking led to a reduction in polyphenolic content in all samples, ranging from a minimum of 1.5-fold decrease for AF3c to a maximum of 2.5-fold decrease for AP6c, resulting in about a 60% loss compared to the raw samples. As documented in previous studies [43], the decline in TPC in cooked pasta extracts may be due to the degradation of polyphenols during boiling or solubilisation, primarily of the free fraction, into the cooking water. To counteract this decrease, it is advised to reduce cooking time, favouring pasta “al dente.” The lower loss for the extracts of pasta with pulp than peel reflects the different polyphenolic compositions of the two apple parts. Our previous work [20] revealed that the flesh polyphenol extract is particularly rich in catechin and epicatechin, while the peel extract contains more procyanidin B2, phloretin glucoside, hyperoside, phloridzin, and anthocyanins than the pulp. These specific polyphenols contribute to the overall polyphenol content and health benefits of apple pulp and peel. Finally, by applying a *t*-test, a commonly used statistical method for mean comparison, we determined that TPC values in all four fortified pasta variants were significantly higher than the control (*p* < 0.05).

### 3.3. Evaluation of the In Vitro Inhibitory Potential of Enriched Pasta on Digestive Enzymes

Before analysing enriched pasta samples, we first evaluated the impact of *Annurca* apple pulp and peel extracts on key enzymes involved in lipid and glycaemic metabolism [44]. In vitro assays demonstrated concentration-dependent inhibition of these enzymes, with IC50 values presented in Table 3. As depicted in Table 3, both extracts showed significant potential for metabolic regulation, with peel extracts exhibiting stronger inhibitory activity against *α*-amylase and *α*-glucosidase, key enzymes in starch digestion, compared to flesh extracts. By delaying the action of these enzymes, postprandial blood glucose levels can be better controlled, offering a practical approach to managing the glycaemic response [45].

This finding aligned with the specific context and relevance observed in comparison with the Monti Sibillini Rosa apple [46].

Similarly, *Annurca* extracts inhibited pancreatic lipase and CEase, enzymes responsible for lipid hydrolysis. While peel extracts were more effective against *α*-glucosidase and amylase, pulp extracts showed greater inhibition of lipase and CEase activities, consistent with the findings by Tenore et al. [47]. These differences reflect the distinct polyphenolic profiles of apple flesh and peel, with apple peels rich in polyphenols, which play a crucial role in protecting the fruit from environmental stressors. Notably, peel extracts are particularly rich in compounds like phloridzin, associated with hypoglycaemic effects [48], and procyanidins, linked to lipid metabolism regulation [49]. The observed synergistic action of these polyphenols further enhances their inhibitory effects on digestive enzymes. Differences in enzyme structure, catalytic mechanisms, and substrate specificity may explain the varying degrees of inhibition. Our in vitro studies confirmed the extracts' capacity to inhibit these enzymes.

**Figure 2:**
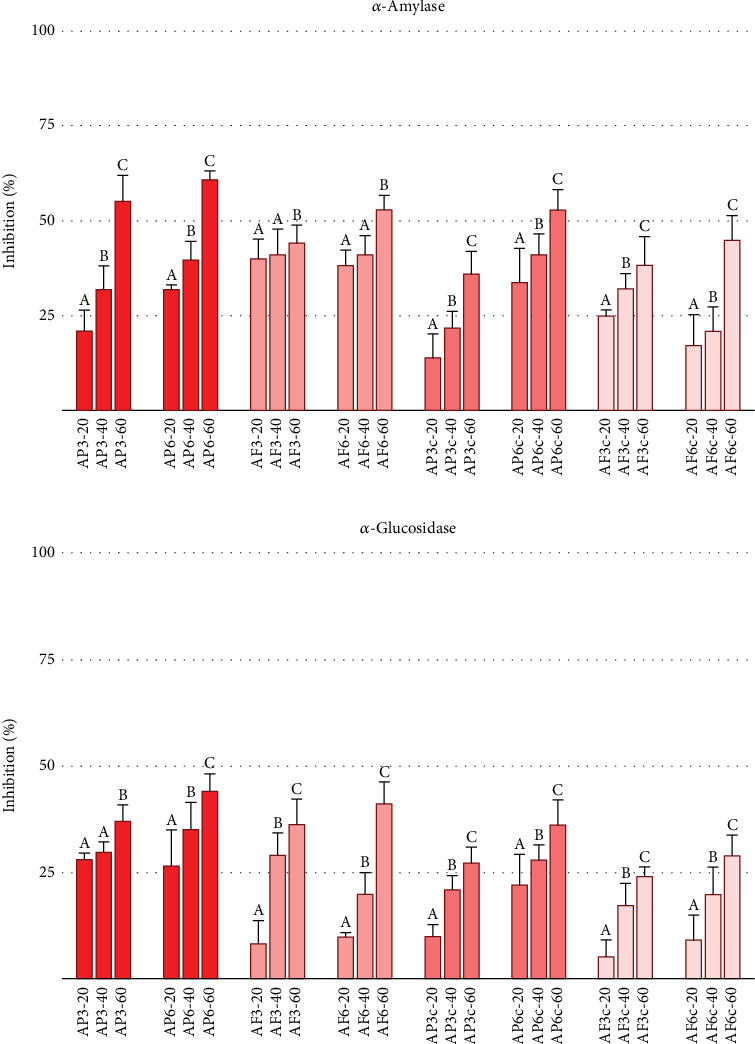
Inhibition of *α*-amylase (a) and *α*-glucosidase (b) enzyme activities. Inhibition performed by extracts obtained from peel-enriched pasta (AP3, AP6), pulp-enriched samples (AF3, AF6), and the respective samples after cooking (AP3c, AP6c, AF3c, and AF6c). All values are mean ± SD, *n* = 3. On the *X*-axis, the three doses used are as follows: 20-40-60 mg/mL. Different letters indicated significant differences among concentration groups with *p* < 0.05.

**Figure 3:**
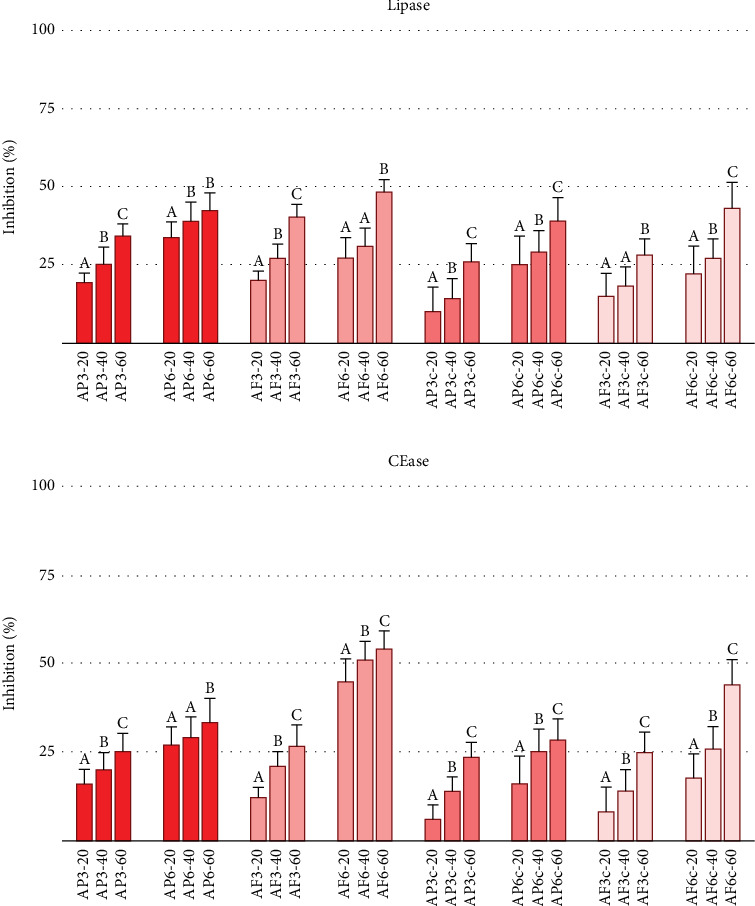
Inhibition of lipase (a) and cholesterol esterase (b) enzyme activities. Inhibition obtained by extracts from peel-enriched pasta (AP3, AP6), flesh-enriched samples (AF3, AF6), and the respective samples after cooking (AP3c, AP6c, AF3c, and AF6c). All values are mean ± SD, *n* = 3. On the *X*-axis, the three doses used are as follows: 20-40-60 mg/mL. Significant differences among concentration groups were indicated with different letters with *p* < 0.05.

Encouraged by these results, we tested the inhibitory properties of enriched pasta samples containing 3% and 6% *Annurca* apple flesh or peel powders. As shown in Figures 2 and 3, the enriched samples inhibited *α*-amylase and *α*-glucosidase activities, as well as lipase and CEase, while control pasta samples showed no activity. This confirms that the bioactivity is attributable to the *Annurca* flours, aligning with previous studies on this cultivar as a rich source of polyphenols with inhibitory capacities. The composition of polyphenolic compounds can indeed be altered by heat treatments such as boiling, a common practice in food preparation that is essential for pasta [50]. We examined whether the enriched pasta retained its inhibitory properties even after cooking. As depicted in Figures 2 and 3, the results indicated that cooking did not eliminate the inhibitory potential of the samples, confirming the robustness of our enriched pasta formulation.

Our previous works and the literature established that tagliatelle enriched with specific percentages of vegetable flour exhibited inhibitory effects on various digestive enzymes [38, 51]. However, the lower inhibitory properties observed in cooked samples, potentially due to the polyphenol loss in cooking water and thermal degradation of phenolic compounds, necessitate further investigation. This area of study, which has also attracted the interest of other researchers [52, 53], requires immediate attention to understand the underlying mechanisms fully.

### 3.4. Predictive GI

To further explore the inhibition of starch-digesting enzymes by the enriched pasta, an in vitro digestion of cooked samples was performed by monitoring starch degradation levels at set times and calculating the expected GI of the four formulations. The in vitro digestion provides valuable insights and serves as an appropriate analytical system for studying the properties of new formulations [37]. Indeed, when adding other components to pasta, it is essential to verify the functional ability of enriched samples, considering that the stability of the matrix could be compromised by these ingredients and potentially create structural pores. In this context, pasta is a low GI food, unlike other grain-based foods such as rice with high GI, owing to its unique structure and accessibility to digestive enzymes. Indeed, the mechanisms of starch digestion are complex and do not solely hinge on the protein–starch matrix peculiarities or the structural state of the starch [16]. As shown in Table 4 for all samples, pasta supplementation at 3% or 6% *Annurca* flours reduced starch digestibility, yielding lower pGI values compared to control pasta, indicating that apple powder components interfered with and inhibited the action of amylase and glucosidase enzymes. In this study, *Annurca* powder additions altered the pGI of pasta samples. In line with the inhibition tests shown in Figure 2, AP3c returned a slightly lower pGI value (42.54) compared to AF3c (45.76). However, doubling the enrichment percentage did not result in a significant difference between the two formulations.

Our findings have practical implications. The addition of 3% and 6% *Annurca* powder to pasta can yield products with lower pGI than traditional pasta, a significant development for diabetic patients and individuals seeking healthier dietary options. A potential limitation of the use of apple flour in pasta production is its colour. However, while some consumers prefer the traditional yellow colour of pasta, others may see the brown hue of enriched pasta as a sign of healthier, more natural ingredients. Our research emphasizes the significant but often underappreciated potential of incorporating apple peels into food production. While the pulp is commonly used for its nutritional benefits, apple peels are packed with phytochemicals that possess remarkable bioactive properties. Regrettably, large quantities of peels are discarded during the manufacturing of apple sauce, apple juice, or canned apples. By promoting the incorporation of apple peel in pasta and raising awareness of its benefits, we can take meaningful steps toward encouraging the consumption of more sustainable foods, an increasingly critical issue today. The complex phytochemical composition and the synergistic interaction among these compounds were key to the observed enzymatic inhibition. The high phytochemical content in apple peel extracts strongly supports their application in these innovative uses.

Numerous studies have shown that polyphenols inhibit enzymes like *α*-amylase, glucosidase, lipase, and CEase, contributing to hypoglycaemic effects and benefits in obesity [54]. Despite these findings, the molecular mechanisms and factors governing the functions and effects of polyphenols remain unclear. This underscores the importance of further research to fully reveal the health and nutritional potential of polyphenols.

Overall, pasta with *Annurca* peel and flesh flour achieved a reduction in pGI comparable to some vegetable-enriched formulations but less effective than legume-based alternatives, which showed the most significant impact on glycaemic response. For instance, pasta made from chickpeas had a GI of 36, making it a lower GI pasta choice. Other legume-based pasta (e.g., lentils and faba beans) showed a significantly lower pGI due to its high protein and fiber content. Some formulations, such as those with 100% legume flours, achieved GI values as low as 20–23, while blends with 25%–35% legume flour reduced the GI substantially (from 72 to 40) [55, 56].

Vegetable flour–enriched pasta offers varying reductions in pGI. While chard, purslane, and onion flours showed significant effects, chicory flour did not [38, 51, 57]. Persimmon flour at low concentrations (3%) reduced the pGI slightly (from 85 to 79, about 7%), but higher amounts (6%) led to an increase [58]. Okara flour–enriched pasta strongly reduced pGI (~45%) due to its impact on starch hydrolysis but also decreased protein digestibility due to antinutrients [59].

The latter cited studies did not examine lipase and CEase inhibition or their potential effects on lipid metabolism. In addition, while pasta enriched with small amounts of vegetable flour retains the classic taste of wheat pasta with subtle flavor variations, legume-based pasta undergoes a more pronounced transformation in flavor, texture, and cooking behavior. This makes it a valuable nutritional alternative with a distinctly different taste profile.

Our study highlights the potential of *Annurca*-fortified pasta as a food with health benefits, including its lipid-lowering and glucose-controlling effects.

## 4. Conclusions

Obesity and Type 2 diabetes are prevalent metabolic diseases with an alarmingly increasing incidence [60]. Both conditions are closely linked to sedentary lifestyles and unhealthy dietary habits, with many studies indicating a correlation between their incidence and inadequate fruit and vegetable intake [61]. Consequently, the fortification of popular foods with beneficial natural ingredients has attracted considerable interest from nutrition experts, as their addition to food production promotes well-being by reducing disease risk [57]. In this study, we investigated the *Annurca* apple variety, a historical apple cultivated since the time of the Roman Empire. We thoroughly assessed the total phenolic content and bioactivity of *Annurca* apple flour, examining the inhibitory effects of its peel and pulp derived from dried apples on key enzymes such as *α*-amylase, *α*-glucosidase, lipase, and CEase. Furthermore, we recorded lower pGI values compared to conventional pasta. Our findings suggest that incorporating 3% and 6% *Annurca* powder into pasta can produce products with a lower pGI than traditional pasta, offering potential benefits for diabetic patients and those seeking healthier dietary options. However, further research is needed to evaluate consumer acceptance, investigate in vivo glycaemic effects, and validate the physiological impact observed in vitro. Additionally, studies should focus on improving processing techniques to preserve the maximum amount of phytonutrients in pasta and elucidating the mechanisms by which apple powder influences the nutritional properties of food products. This research presents exciting opportunities for the pasta industry to respond to growing consumer demand for healthier options, with *Annurca*-enriched pasta potentially becoming a key part of fat and glucose-controlled diets, benefiting human health.

## Figures and Tables

**Table 1:** Cooking performance of pasta samples.

**Sample**	**Optimal cooking time (min)**	**Cooking loss (g/100 g)**	**Swelling index (g water/g dry pasta)**	**Water absorption index (g/100 g)**
*Ctrl*	10^a^	4.5 ± 0.28^a^	1.85 ± 0.07^a^	94.0 ± 3.2^a^
*AP3c*	10^a^	4.6 ± 0.2^a^	1.65 ± 0.04^a^	91.1 ± 4.7^a^
*AP6c*	10^a^	5.2 ± 0.4^a^	1.84 ± 0.08^a^	93.0 ± 3.3^a^
*AF3c*	10^a^	4.9 ± 0.42^a^	1.50 ± 0.10^a^	80.5 ± 4.1^a^
*AF6c*	10^a^	5.1 ± 0.42^a^	1.55 ± 0.09^a^	86.0 ± 5.9^a^

*Note:* Results were mean values of three measurements ± standard deviation (SD). Equal letters over the columns indicate not statistically significant differences (*p* > 0.05).

**Table 2:** TPC of *Annurca* peel and flesh flours and sample pasta.

**Sample**	**TPC *μ*g GAE/g**	**±SD**
*Peel flour*	1841.4	±35.2
*Flesh flour*	1233	±24.3
*Ctrl*	34.0	±4.1
*AP3*	103.4	±5.1
*AP6*	200.6	±6.9
*AF3*	68.5	±3.9
*AF6*	119.9	±4.2
*AP3c*	54.7	±6.2
*AP6c*	79.8	±5.1
*AF3c*	45.6	±4.3
*AF6c*	51.7	±3.4

*Note:* Results are means of three experiments ± standard deviation (SD). Ctrl = pasta with durum wheat semolina; AP3 and AP6 = 3%–6% peel powder–fortified pasta; AF3–AF6 = 3%–6% flesh powder–enriched samples; AP3c-AP6c-AF3c-AF6c = cooked pasta samples. All mean values of samples were significantly different from Ctrl, *p* < 0.05 by *t*-test.

Abbreviation: TPC = total phenolic content.

**Table 3:** IC50 values of *Annurca* peel and flesh extracts to inhibit *α*-amylase, *α*-glucosidase, lipase, and cholesterol esterase.

**Sample**	**IC50 CEase ± SD**	**IC50 lipase ± SD**	**IC50 amylase ± SD**	**IC50 glucosidase ± SD**
*Annurca* flesh extract	17.6 ± 0.6 (a)	4.3 ± 0.5 (a)	17.3 ± 4.2 (a)	40.3 ± 4.2 (a)
*Annurca* peel extract	22.2 ± 0.4 (b)	7.3 ± 0.3 (b)	7.2 ± 2.1 (b)	27.5 ± 4.2 (b)

*Note:* IC50: Concentration of sample (mg/g of dry weight) required to inhibit 50% of enzyme activity. Data were mean values of triplicate measurements ± standard deviation (SD). Different letters over the columns indicate statistically significant differences (*p* < 0.05).

**Table 4:** Predicted glycaemic index (pGI) of pasta samples.

**Sample**	**PGI**	**±SD**
*Ctrl*	56.33^a^	±2.2
*AP3c*	42.54^b^	±0.4
*AP6c*	42.37^b^	±2.1
*AF3c*	45.76^b^	±3.7
*AF6c*	41.25^b^	±1.4

*Note:* Data are means of three experiments ± standard deviation. Predicted glycaemic index (pGI) of samples with 3% of peel powder (AP3c), 6% of peel powder (AP6c), 3% of flesh powder (AF3c), and 6% of flesh powder (AF6c). Nonidentical lowercase letters in the same column evidence significant differences (*p* < 0.05).

## Data Availability

The data supporting this study's findings are available on request from the corresponding author.
